# Methyleugenol Has an Antidepressant Effect in a Neuroendocrine Model: In Silico and In Vivo Evidence

**DOI:** 10.3390/ph16101408

**Published:** 2023-10-04

**Authors:** Mayara Cecile Nascimento Oliveira, Ikla Lima Cavalcante, Alana Natalícia de Araújo, Aline Matilde Ferreira dos Santos, Renata Priscila Barros de Menezes, Chonny Herrera-Acevedo, Natália Ferreira de Sousa, Jailane de Souza Aquino, José Maria Barbosa-Filho, Ricardo Dias de Castro, Reinaldo Nóbrega Almeida, Luciana Scotti, Marcus Tullius Scotti, Mirian Graciela Da Silva Stiebbe Salvadori

**Affiliations:** 1 Laboratory of Psychopharmacology, Institute for Research in Drugs and Medicines, Federal University of Paraíba, João Pessoa 58051-900, PB, Brazileualineferrer@gmail.com (A.M.F.d.S.); rcastro@ccs.ufpb.br (R.D.d.C.);; 2 Laboratory of Cheminformatics, Institute for Research in Drugs and Medicines, Federal University of Paraíba, João Pessoa 58051-900, PB, Brazil; 3 Laboratory of Experimental Nutrition, Department of Nutrition, Federal University of Paraíba, João Pessoa 58051-900, PB, Brazil; 4 Department of Pharmaceutical Sciences, Institute for Research in Drugs and Medicines, Federal University of Paraíba, João Pessoa 58051-900, PB, Brazil

**Keywords:** phenylpropanoid, dopaminergic, α1 adrenergic, serotonergic, in vivo, in silico

## Abstract

Major depressive disorder is a severe mood disorder characterized by different emotions and feelings. This study investigated the antidepressant activity of the phenylpropanoid methyleugenol (ME) in adult female mice exposed to a stress model induced by dexamethasone. The animals were randomly divided into groups containing eight animals and were pre-administered with dexamethasone (64 μg/kg subcutaneously). After 165 and 180 min, they were treated with ME (25, 50 and 100 mg/kg intraperitoneally) or imipramine (10 mg/kg intraperitoneally) after 45 min and 30 min, respectively; they were then submitted to tests which were filmed. The videos were analyzed blindly. In the tail suspension test, ME (50 mg/kg) increased latency and reduced immobility time. In the splash test, ME (50 mg/kg) decreased grooming latency and increased grooming time. In the open field, there was no statistical difference for the ME groups regarding the number of crosses, and ME (50 mg/kg) increased the number of rearing and time spent in the center. Regarding in silico studies, ME interacted with dopaminergic D1 and α1 adrenergic pathway receptors and with tryptophan hydroxylase inhibitor. In the in vivo evaluation of the pathways of action, the antidepressant potential of ME (50 mg/kg) was reversed by SCH23390 (4 mg/kg intraperitoneally) dopaminergic D1 receptor, Prazosin (1 mg/kg intraperitoneally) α1 adrenergic receptor, and PCPA (4 mg/kg intraperitoneally) tryptophan hydroxylase inhibitor. Our findings indicate that ME did not alter with the locomotor activity of the animals and shows antidepressant activity in female mice with the participation of the D1, α1 and serotonergic systems.

## 1. Introduction

Major depressive disorder (MDD) is a mood disturbance influenced by different emotions and feelings. Typically, MDD may feature feelings of sadness and helplessness, reduced or lost interest in daily activities (anhedonia), lack of motivation, depressed mood, irritability, and even suicidal ideation [1,2].

The World Health Organization (WHO) reported that in 2017 there were about 322 million people with depression worldwide (World Health Organization, 2017). The first year of the COVID-19 pandemic intensified the prevalence by 25% and the records show that, by 2030, depression is projected to become the most prevalent disease globally, becoming the main cause of population loss among all health problems (WHO 2022). Depression is about twice as prevalent in women than in men, which may be related to increased sensitivity to stress, hormonal variations, and genetic or social factors [3,4]. It is known that stress causes changes that may favor the development of depression [5]; stress is an important physiological response so that when exposure is high, hyperactivity of the hypothalamic-pituitary-adrenal axis (HPA) occurs [6], which stimulates the paraventricular nucleus of the hypothalamus to release corticotropins, which in turn promotes the production of adrenocorticotropic hormone that acts on the adrenal gland and allows the release of cortisol, which is the main stress response hormone and acts on numerous glucocorticoid receptors [7,8]. Hyperactivity of the HPA axis negatively affects the brain’s noradrenergic, serotonergic, and dopaminergic systems, which play crucial roles in regulating mood, attention, and various cognitive functions [9,10]. It is known that one of the causes identified for the development of depression is the reduction of monoamines (noradrenaline, serotonin, and dopamine) in the central nervous system [11,12,13]. Thus, the antidepressants used in the clinic make it possible to reduce antidepressant symptoms through mechanisms that increase the concentration of monoaminergic neurotransmitters, through the action of blocking reuptake and/or monoaminergic receptors [11,12].

Treatment for MDD includes behavioral interventions/self-care strategies, psychotherapy, and pharmacotherapy. Antidepressant drugs are considered essential to treat moderate and severe depression. However, antidepressants do not have a favorable short-term outcome (a latency period is required for a satisfactory effect to occur), and adverse effects may include cognitive and motor changes and increased vulnerability of individuals to become tolerant and dependent [13]. About 30% of patients do not achieve remission [14]. Therefore, developing new effective antidepressants is important.

An alternative for researching new drugs involves products from medicinal plants, especially those producing essential oils, which constitute a large and diverse deposit of pharmacologically active compounds [15].

Plants such as *Ginkgo biloba* L. [16], *Alpinia zerumbet* (Pers.) BLBurtt & RMSm. [17], and *Lafoensia pacari* A.St.-Hil. [18] showed antidepressant activity in animal models and are mainly composed of phenylpropanoids, such as trans-anethole [19] and methyleugenol [20,21]. A study by [22] found that the phenylpropanoid eugenol (present in clove oil) generated antidepressant activity similar to that of imipramine in the behavioral tests of forced swim and tail suspension in mice.

Methyleugenol (ME, 1,2-dimethoxy-4-prop-2-en-1-ylbenzene) (an analog of the phenolic compound eugenol) is a phenylpropanoid that can be extracted from essential oils of plants, such as *Melissa officinalis* L. and *Ocimum basilicum* L. Previous studies revealed that methyleugenol has antinociceptive and anti-inflammatory pharmacological properties [23,24]. A study verified antidepressant activity using [25] male rats. Thus, the present study is the first to evaluate the antidepressant potential of phenylpropanoid methyleugenol in vivo using female mice and the possible pharmacological targets of these findings through in silico computational prospecting.

## 2. Results

### 2.1. Behavioral Pharmacology Screening and LD50 Estimation

Under the conditions evaluated, the results showed that during the 14 days of observation, there were no deaths or lasting behavioral changes in the animals treated with a dose of 2000 mg/kg.

The phenylpropanoid ME by i.p. route showed low toxicity, with no evidence of changes in skin, hair, or eyes (a priori in class 5); thus, the LD50 was estimated to be found in values above 2000 mg kg^−1^ and below 5000 mg kg^−1^. In this manner, a range of secure doses could be determined for subsequent in vivo execution.

### 2.2. Tail Suspension Test

**Figure 1:**
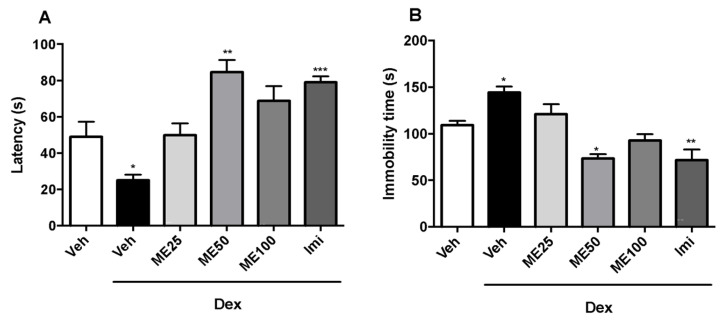
Effect of methyleugenol (ME 25, 50 and 100 mg/kg, i.p.), vehicle (Veh i.p.), dexamethasone (Dex 64 μg/kg s.c.), and imipramine (Imi 10 mg/kg i.p.) on latency to immobility (**A**) and Time to immobility (**B**) in the tail suspension test. Values are expressed as mean ± SEM (n = 8). One-way ANOVA followed by Dunnett’s Test. * *p* < 0.05; ** *p* < 0.01; *** *p* < 0.001 versus Vehicle (Veh).

During the evaluation of latency to immobility (Figure 1A), results of one-way ANOVA revealed that acute treatment with ME (at a dose of 50 mg/kg [84.7 ± 6.6]) and the reference antidepressant (imipramine [Imi] [79 ± 3.1]) showed higher latency to immobility. The group of animals receiving dexamethasone (Dex) (25 ± 3.1) had their latency reduced when compared to the group receiving vehicle (Veh) (49.8 ± 6.5), (F (5, 42) = 12.51; *p* < 0.0001). Regarding immobility time, animals treated with ME 50 mg/kg (73.2 ± 4.7) and Imi (71.7 ± 11.3) reduced this parameter, while the dexamethasone (Dex) group (144.1 ± 6.5) increased this parameter compared to the Veh group (109.1 ± 4.7) [F (5, 42) = 12.7; *p* < 0.0001] (Figure 1B).

### 2.3. Splash Test

**Figure 2:**
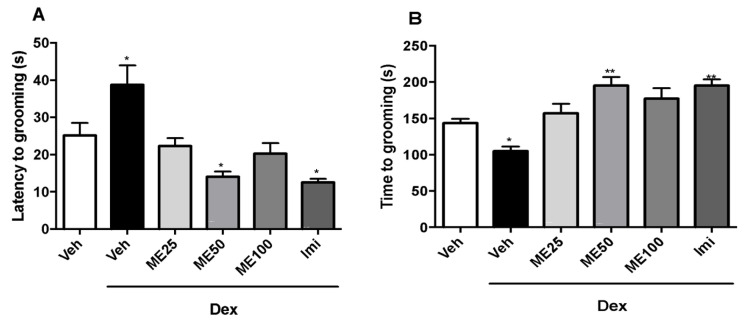
Effect of ME (25, 50 and 100 mg/kg i.p.), vehicle (Veh) (i.p.), dexamethasone (Dex) (64 μg/kg s.c.) and imipramine (Imi) (10 mg/kg i.p.). (**A**) Latency to grooming and (**B**) Grooming time in the Splash Test. Values expressed as mean ± SEM (n = 8). One-way ANOVA followed by Dunnett’s Test. * *p* < 0.05, ** *p* < 0.01 versus Vehicle (Veh).

Methyleugenol in the intermediate dose (50 mg/kg) (22.2 ± 2.1) and imipramine (Imi) (12.5 ± 0.9) decreased the latency in seconds for grooming (Figure 2A) and the dexamethasone (Dex) group (38.7 ± 5.1) increased this parameter compared to the vehicle (Veh) group (25.1 ± 3.3), (F (5, 42) = 10.0; *p* < 0.0001). The grooming time (Figure 2B) increased in the groups treated with ME at a dose of 50 mg/kg (195 ± 11.9) and imipramine (Imi) (195.3 ± 8.4), while the dexamethasone (Dex) group (105.1 ± 5.9) had a reduction in this parameter compared to the vehicle (Veh) group (143.8 ± 5.7) (Figure 2B) [F (5, 42) = 10.7; *p* < 0.0001].

### 2.4. Open Field Test

**Figure 3:**
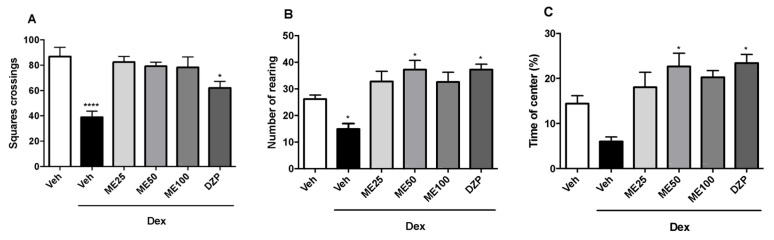
Effect of ME (25, 50 and 100 mg/kg i.p.), vehicle (Veh) (i.p.), dexamethasone (Dex) (64 μg/kg s.c.) and imipramine (Imi) (10 mg/kg i.p.) on the number of crosses (**A**); Rearing (**B**); Time of center (**C**) in Open Field Test. Values expressed as mean ± SEM (n = 8). One-way ANOVA followed by Dunnett’s Test. * *p* < 0.05; **** *p* < 0.0001 versus Vehicle (Veh).

Figure 3 demonstrates the outcomes derived from the open field assessment. In the evaluation of the number of crossings in the arena (Figure 3A), the group treated with dexamethasone (Dex) (38.8 ± 4.7) and diazepam (DZP) (61.8 ± 5.2) showed a reduction in the number of crossings and differed from the vehicle group (Veh) (86.6 ± 7.4) (F (5, 42) = 9.3; *p* < 0.0001).

Regarding the rearing number (Figure 3B), animals treated with ME at the intermediate dose (50 mg/kg) (37.2 ± 3.4) and diazepam (DZP) (37.2 ± 2.0) performed this behavior more than the dexamethasone (Dex) group (14.8 ± 2.1) compared to the vehicle (Veh) group (26.1 ±1.6) (F (5, 42) = 8.2; *p* < 0.0001).

Regarding the percentage of time that the animals remained in the center of the arena (Figure 3C), treatments with ME 50 mg/kg (22.6 ± 2.9) and diazepam (DZP) (23.3 ± 1.9) increased this permanence in the center of the open field, while those treated with dexamethasone (Dex) (6.0 ± 1.1) reduced this percentage compared to the vehicle (Veh) group (14.3 ± 1.8) (F (5, 42) = 8.5; *p* < 0.0001).

### 2.5. Assessment of the Mechanisms of Action in the Antidepressant Effects of Methyleugenol

**Figure 4:**
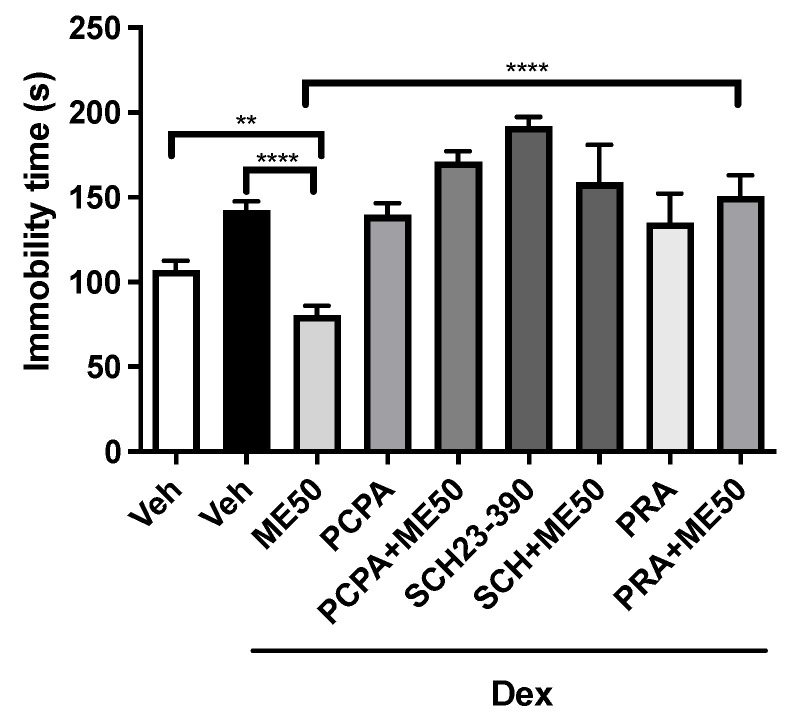
Assessment of the mechanisms of action involved in the antidepressant activity of methyleugenol (ME 50 mg/kg, i.p.) on immobility time in TSC. Vehicle (Veh i.p.), dexamethasone (Dex) (64 μg/kg s.c.); PCPA (100 mg/kg, i.p.); SCH23390 (1 mg/kg s.c.); prazosin (PRA) (1 mg/kg i.p.). Values expressed as mean ± SEM (n = 8). One-way ANOVA followed by Tukey’s test. ** *p* < 0.01; **** *p* < 0.0001 versus ME50.

When investigating the pharmacological pathways, we found that all groups, Veh (107.3 ± 5.3), DEX (141.3 ± 6.5), PCPA (139.8 ± 6.7), PCPA + ME (171.2 ± 5.9), SCH2330 (191.9 ± 5.4), SCH23390 + ME50 (158.8 ± 4. 5), prazosin (PRA) (134.9 ± 3.9), and PRA + ME50 (150.8 ± 4.4), increased immobility time compared to the ME50 group (80.5 ± 5.4), which had this time reduced. A previous administration of PCPA (serotonin synthesis inhibitor), SCH-23390 (D1 antagonist), and prazosin (α1-adrenergic antagonist) prevented the antidepressant-like effect of both ME (F (8, 65) = 37.3; *p* < 0.0001) (Figure 4).

### 2.6. In Silico Methodologies

To investigate whether the D1 dopaminergic and α1 adrenergic pathways are involved in the antidepressant impact of methyleugenol, we carried out a study using molecular docking. Through this study, it is possible to assess the probability of interaction between ME and the active site of the selected proteins. The interaction probability can be analyzed through the MolDock score, which calculates the interaction energy, where the higher the stability of the interaction, the lower the energy value. We also analyzed it through intermolecular interactions between ME and amino acid residues present in the binding site of proteins. We validated the docking analysis through redocking and RMSD calculation.

Table 1 shows the MolDock score energy values for ME of the three proteins, as well as of the molecules SCH 23390 inhibitor of the dopaminergic D1 pathway, prazosin inhibitor of the α1 adrenergic pathway, and PCPA inhibitor of the tryptophan hydroxylase pathway, and also the compounds crystallized in the binding site of each enzyme: fenoldopam (catechol agonist of the D1 dopaminergic pathway), (+)-cyclazosin (an inverse agonist of the α1 adrenergic pathway), and LP533401 (inhibitor of the tryptophan hydroxylase pathway). For a fair comparison between the energy values for every compound within the binding site of each enzyme, the measurements were also standardized according to the value of their molecular masses. Table 1 also provides the RMSD value for the crystallized ligand of each protein.

## 3. Discussion

In the present study, we investigated and confirmed the antidepressant psychopharmacological profile of the phenylpropanoid methyleugenol using in vivo behavioral and in silico computational methodologies.

The concept that products obtained from natural origin possess important pharmacological properties is widely spread by the scientific community and the population. However, it is important to be aware of the possibility that they present toxic effects that are often overlooked [26]. Pharmacological screening qualitatively assesses their actions on the central nervous system (CNS) and autonomic nervous system (ANS) and estimating the acute toxicity of any substance is of utmost relevance to ensure the safety of its use. Methyleugenol at a dose of 2000 mg/kg caused behavioral changes suggestive of drugs with CNS activity but without causing toxic effects and lethality. This estimate of LD50 allowed us to evaluate the doses that were used in the behavioral tests, free of toxic effects.

The antidepressant effect of methyleugenol was investigated in a model of depression induced by dexamethasone applied i.p. [27]. Major depressive disorders have been linked to dysregulation of the hypothalamic-pituitary-adrenal (HPA) axis, which involves the secretion of glucocorticoids in excess [28], and persistent activation of its receptors by high levels of glucocorticoids can cause neural damage, with molecular and behavioral alterations, resulting in MDD and other neuropsychiatric disorders [29]. Patients treated with glucocorticoids have exhibited psychiatric and cognitive symptoms similar to depression. Already in animal models, chronic and acute glucocorticoid administration caused behavioral modifications similar to those found in humans with the disorder [30,31]. Thus, in this animal model of inducing depressive-like behavior, it is possible to mimic symptoms associated with the disease and develop pathophysiological aspects analogous to the human condition where treatment with antidepressant drugs can reverse the changes in the model [32,33].

Besides the choice of model induction that induces depressive-symptomatic behavior, the tests chosen to evaluate this behavior are also important. Indeed, the tail suspension test is one of the most widely used tests for screening antidepressant drugs [34] and is considered sensitive and specific for most classes of antidepressant drug [35]. Thus, the animal placed in an inescapable situation (trapped by the tail) develops a posture of immobility after escape attempts, and we perceived this as giving up, which characterizes a depressive state. The antidepressant substances analyzed can reverse such a condition by reducing the time of immobility [36,37]. Methyleugenol (at a dose of 50 mg/kg) increased latency in the tail suspension test, showing an effect analogous to that of imipramine and showing the antidepressant-similar activity of the substance. Although little evaluated, the verification of latency contributes to further understanding of the effects of drugs, as certain classes of antidepressants alter this parameter [38].

In 2014, Li and collaborators conducted a study using the Dex protocol, in which Dex was administered during the neonatal period (1 to 3 days) in doses ranging from 0.1 to 0.5 mg/kg body weight. Despite differences in the protocol when compared to the one used in this study, Dex increased the immobility time of the animals in the tail suspension test when compared to the control group, confirming the results obtained in this study. Still validating our data, [39] found the same result as the previous authors when administering a higher dose of Dex (1.5 mg/kg) in rats. Recently, an increase in immobility time was observed in male mice submitted to single-dose injections of Dex (64 μg/kg) in the forced swim test [31,40].

To corroborate our findings in the tail suspension test, we performed the splash test. Animals submitted to this test exhibit a shorter grooming time, which is interpreted as anecdotal behavior. In contrast, administering classical antidepressants increases the time spent on this behavior [41,42]. In turn, methyleugenol (intermediate dose 50 mg/kg) decreased the latency to grooming as well as increased the total grooming time, again showing an antidepressant-similar effect in female mice and similar to the standard imipramine. In a study similar to ours, acute treatment of a single dose of dexamethasone increased immobility time in the tail suspension test, inducing depressive behavior in animals, which was counteracted with venlafaxine, a conventional antidepressant [43].

The open field test is widely used for assessing spontaneous locomotor activity and provides clues to the emotional state of rodents [44,45,46]. In our study, in the open field test, only the animals that received the positive control (diazepam) and the dexamethasone group reduced the number of crosses. Methyleugenol (50 mg/kg) and animals receiving diazepam increased the number of rearing and dwell time in the center, related to Dex. A methyleugenol dose of 25 mg/kg decreased the latency to rearing. This set of results suggests that methyleugenol did not alter spontaneous locomotor activity and showed an effect indicative of anxiolytic activity, providing clues to the emotional state of the mice.

Once we identified the antidepressant-similar activity of ME in female mice, we needed to understand how this action occurs. Accordingly on the principles of the 3Rs (Replacement, Reduction, and Refinement) in non-clinical research (Russel and Burch, 1992), we conducted the study in silico to evaluate in vivo by which route(s) the ME was exerting the antidepressant effect.

**Figure 5:**
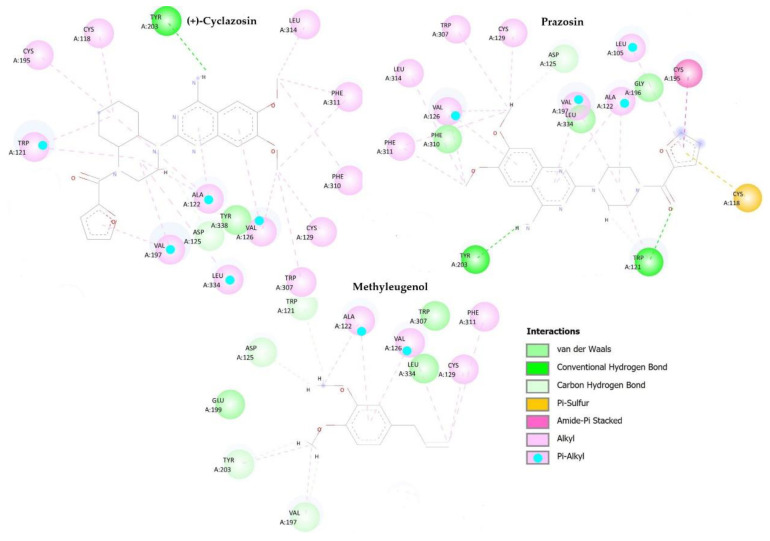
Molecular interactions between the compounds (+)-cyclazosin, prazosin, and methyleugenol, and active site’s amino acid residues of the α1 adrenergic protein (PDB ID: 7B6W).

In molecular docking studies, we found interaction probabilities between methyleugenol and receptors of the D1, α1 adrenergic, and tryptophan hydroxylase pathways. The α1 adrenergic protein (PDB ID 7B6W) obtained from PDB Data Bank is crystallized with the inverse agonist (+)-cyclazosin. The analysis focused on the binding site, examining the following amino acid residues: Trp 121, Asp 125, Val 126, Cys 129, Val 197, Tyr 203, and Phe 311 were found to be the most influential for enzyme activity.

(+)-cyclazosin makes hydrogen interactions with Tyr 203, while forming van der Waals interaction with Asp 125. Additionally, it establishes Alkyl-type interactions with Val 126, Phe 311, Val 127, Trp 121, and Cys 129 amino acid residues. The antagonist Prazosin participates in hydrogen interactions with Tyr 203 and Trp 121, and carbon–hydrogen bond-type interactions with Asp 125. Alkyl-type interactions emerge with Phe 311, Val 197, Val 126, and Cys 129 residues. Methyleugenol establishes carbon–hydrogen bonding interactions with Val 197, Tyr 203, Asp 125, and Trp 121, while also forming Alkyl-type interactions with Val 126, Phe 311, and Cys 129. The visual representation of these interactions between these molecules and the protein’s binding site is displayed in Figure 5.

These interactions explain the energetic behavior of the connections within the compound and the binding site of the α1 adrenergic protein. Prazosin had the strongest interaction, followed by methyleugenol and (+)-cyclazosin. Imipramine was not evaluated for its interaction with any of the proteins in this study as its mode of operation involves inhibiting the reuptake of serotonin and noradrenaline, rather than influencing these proteins.

The dopaminergic D1 receptor (PDB ID 7CKW), obtained from the PDB Data Bank, is crystallized with the catechol agonist fenoldopam. According to the literature, the active site of D1 receptor agonists and antagonists act on the same active site, and residues Asp103, Trp 321, Phe 289, Phe 288, Trp 285, Asn 292, and Ser 202 are considered the most influential for antagonist activity at the D1 receptor [47,48].

The interactions occurring between the agonist fenoldopam and the active site’s amino acid residues of the D1 receptor are stronger than the interactions between the antagonists SCH 23390 and methyleugenol. Fenoldopam makes hydrogen-type interactions with the Ser 202; engages in van der Waals contacts with residues Asp 103, Asn 292, Trp 321, Phe 289 and Trp 285; and Pi-Pi interaction with the amino acid residue Phe 288.

**Figure 6:**
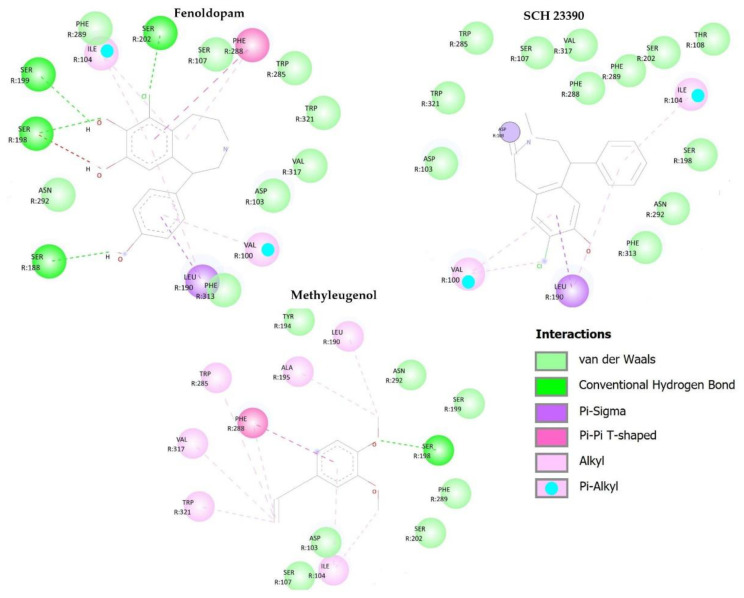
Molecular interactions between the compounds fenoldopam, SCH 23390, and methyleugenol and active site’s amino acid residues of the dopaminergic protein D1 (PDB ID: 7CKW).

Both the control antagonist used in this study (SCH 23390) and the target molecule (methyleugenol) share similarities in interactions with active sites’ amino acid residues within the D1 receptor. Both perform van der Waals interactions with residues Asp 103, Asn 292, Phe 289, and Ser 202. The SCH 23390 also performs van der Waals interactions with residues Phe 288, Trp 285, and Trp 321, while methyleugenol performs alkyl or Pi-Pi type interactions with residues Trp 321, Trp 285, and Phe 288. The similarities between the antagonists are also observed in the interaction energy (Table 1). Figure 6 shows the interactions between these molecules and the D2 receptor.

The tryptophan hydroxylase protein (PDB ID 7ZIK) obtained from the PDB dataBank is crystallized with the LP53340 inhibitor. Based on the analysis of the active site, the amino acid residues Thr 265, Glu 317, Tyr 235, Ser 336, Ser 337, Gly 333, and Arg 257 were considered the most influential to inhibit the action of the protein [49].

**Figure 7:**
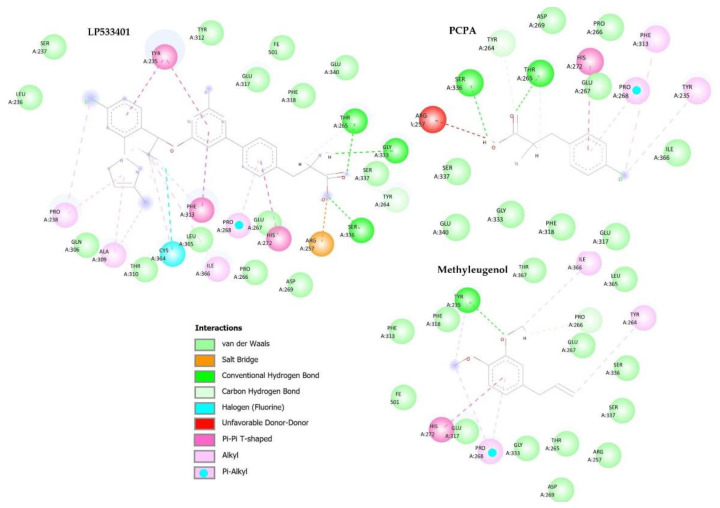
Molecular interactions between the compounds LP533401, PCPA, and methyleugenol, and active site’s amino acid residues of the tryptophan hydroxylase protein (PDB ID: 7ZIK).

The LP533401 inhibitor crystallized with the protein and the inhibitor used in the in vitro analysis, PCPA, performs hydrogen interactions with amino acid residues Thr 265 and Ser 336, and van der Waals interactions with Glu 317, Ser 337. Methyleugenol interacts with hydrogen only with the amino acid residue Tyr 235, and with the other six amino acid residues considered to be most important, methyleugenol interacts through van der Waals interactions. The LP533401 inhibitor also performs Pi-Pi-type interactions with the Tyr 235 and a salt bridge interaction with the Arg residue 257. In addition, the PCPA inhibitor performs alkyl-type interactions with the Tyr residue 235 and an unfavorable hydrogen interaction with the amino acid residue Arg 257. Figure 7 shows the interactions between these molecules in the active site of the tryptophan hydroxylase protein.

We assessed the possibility of interaction through the noradrenergic system in vivo. Because of their involvement in the pathophysiology of depression, some antidepressants increase the availability of noradrenaline at synapses [50]. α1- and α2-adrenergic receptors are linked to antidepressant drug responses in behavioral models of depression [51,52].

In the in vivo evaluation, methyleugenol had its antidepressant effect blocked by pretreatment with prazosin (α1-adrenergic antagonist), increasing immobility time in the tail suspension test. Thus, we suggest that ME interacts with the α1-adrenergic receptors of the noradrenergic system to exert its antidepressant-similar effect. Similar to our findings, [53] demonstrated that the structural analog of eugenol (α-asarone) exhibited antidepressant-sympathetic activity reversed by prazosin pretreatment.

Another important system implicated in depression is the serotoninergic system. The increase in the level of 5-hydroxytryptamine (serotonin) would be related to the combatting of depression [54]. We used PCPA, a selective inhibitor of serotonin synthesis that inhibits the enzyme tryptophan hydroxylase and depletes the level of serotonin without affecting the levels of noradrenaline and dopamine in the brain. In the present study, PCPA administered alone showed a significant difference regarding immobility time in the tail suspension test compared to methyleugenol. When the animals were pretreated with PCPA and then with methyleugenol, they also increased the immobility time, thus reversing the anti-immobility effect of methyleugenol. Similar to our results, the study conducted by [55] found that pretreatment with PCPA altered the effect on the immobility time of *Carthamus tinctorius* L. in the tail suspension test.

To continue the understanding of the mechanism of action of methyleugenol, we evaluated the possibility that its action is still through the dopaminergic system. This is especially involved in mood regulation [56], and it is known that dysregulation of the mesolimbic and mesocortical dopaminergic pathway is related to signs of melancholy and altered cognition in depression [57,58,59]. In addition, there are already records of the efficacy of antidepressants that act on the dopaminergic pathway [60].

Thus, involvement of the dopaminergic (D1) system in the antidepressant effect of ME was found, as pretreatment of the mice with SCH23390 (D1 receptor antagonist) blocked the effect induced by methyleugenol, thus increasing the immobility time in the tail suspension test. Only the administration of SCH23390 increased immobility, as was also observed in the findings of [35], who by administering SCH23390 to mice, reversed the antidepressant effect of gallic acid.

Our findings obtained in the present study allow us to conclude that the administration of methyleugenol in animals submitted to the dexamethasone-induced depression model produces a relevant antidepressant effect without altering locomotor activity. Its action on depressive-like behavior seems to be associated with the involvement of the D1 dopaminergic, α1 adrenergic, and serotoninergic systems.

## 4. Materials and Methods

### 4.1. Chemicals and Drugs

Methyleugenol (ME) was purchased from Sigma-Aldrich^®^ Chemical (St. Louis, MO, USA), Diazepam (Cristália, Itapira, SP, Brazil), Distilled water (Federal University of Paraíba [UFPB], João Pessoa, PB, Brazil), Dexamethasone (Sigma, St. Louis, MO, USA), Ethanol (Vetec, Duque de Caxias, Brazil), R (+)-SCH23390 Hydrochloride (Sigma, USA), Imipramine (Cristália, Itapira, SP, Brazil), p-chlorophenylalanine (PCPA) (Sigma, USA), Prazosin (Sigma, USA), Sucrose (Vetec, Brazil), Tween 80 (polyoxyethylene sorbitan monoelate, Sigma, USA).

We prepared the substances in their doses minutes before use. The decimal concentrations used allowed the injection of 0.1 mL/10 g of animal weight. Methyleugenol was dissolved in distilled water with the help of tween 80, in doses 25 mg/kg, 50 mg/kg, and 100 mg/kg, for intraperitoneal (i.p.) administration. For the negative control, Tween 80 1% in distilled water (i.p.) was used.

We diluted the other drugs in distilled water or 0.9% saline solution. We administered them i.p. (methyleugenol, saline, imipramine, prazosin, and PCPA) or subcutaneously (s.c.) (dexamethasone and SCH23390).

### 4.2. Animals

Female albino Swiss mice (*Mus musculus*), weighing 25–35 g, approximately 3 months old, were housed in polyethylene cages and kept under controlled temperature (21 ± 1 °C). They had free access to pellet feed (Quimtia^®^) and water available in polyethylene bottles, and were kept under a 12 h light/dark cycle (the light phase from 6 am to 6 pm). We used mice for all experimental protocols. The animals were from the Animal Production Unit (UPA), Institute for Research in Drugs and Medicines (IPeFarM), UFPB. All procedures were conducted according to the guidelines proposed by the National Institutes of Health (NIH) Animal Care and Use Committee and approved by the Animal Ethics Committee (CEUA), UFPB (certificate no. 045/2017).

### 4.3. In Vivo Methodologies

#### 4.3.1. Methyleugenol Acute Toxicity Estimation and Pharmacological Screening

We performed the determination of acute toxicity in the procedures adapted from the Brazilian Health Regulatory Agency [61] and the Organization for Economic Cooperation and Development “Guidelines for testing of chemicals” [62] with modifications.

Groups of Swiss mice (consisting of three mice per group, including the control) were administered a single dose of 2000 mg/kg of ME. The control group received treatment with 0.9% saline + 0.5% Tween (i.p.). Following administration, we conducted observations at intervals of 30 min, 1st hour, 2nd hour, and 4th hour; and subsequently once daily up to the fourteenth day. After the fourteenth day, the animals were weighed and euthanized with an anesthetic overdose injection. From this step, we established the doses from the estimated LD50 value.

#### 4.3.2. Induction of Depressive-like Behavior

To evaluate the antidepressant activity, we initially induced depressive-like behavior in the animals, which was adapted from the studies of Wrobél and colleagues (2014). Dexamethasone (64 μg/kg s.c.) was administered to all groups, except the saline group, 3 h 30 min before we performed the behavioral tests. We ended the experiments by noon because of peak endogenous corticosterone release.

##### Tail Suspension Test

After the time of administration of dexamethasone (180 min our 165 min), we performed the test according to the protocol described previously [63]. Groups of mice were used (n = 8). The groups Vehicle (control group: Tween 80 0.5%), imipramine (30 mg/kg i.p.), and ME (25, 50 and 100 mg/kg i.p.) had their substances administered 30 (imipramine) and 45 (ME) minutes before the beginning of the test. After the treatment period, we suspended the mice 58 cm above the ground with tape placed approximately 1 cm from the tip of the tail. Each animal remained in this position for 6 min. The behavioral parameters observed were latency to immobility and total immobility time. The animal was classified as immobile when it showed no body movement and remained totally still.

##### Splash Test

To analyze the self-cleaning behavior (grooming) of the animals, a 10% sucrose solution was sprayed on the animal’s back. The sucrose solution is thick and induces an unpleasant sensation in the animal, prompting it to engage in body cleaning, a typical self-care action. Animal cleanliness represents a form of self-care behavior, and its decrease is linked to depressive symptoms (10). After administration of dexamethasone, groups of mice were used (n = 8). The groups Vehicle (control group: Tween 80 0.5%), imipramine (30 mg/kg i.p.), and ME (25, 50 and 100 mg/kg i.p.) had their substances administered 30 (imipramine) and 45 (ME) minutes before the beginning of the test. The animal was individually placed inside an acrylic box (9 × 7 × 11 cm) and the latency parameters for the onset of self-cleaning and the time the animal remained in the self-cleaning behavior were observed for 5 min. Between observations, we sanitized the apparatus with 10% alcohol.

##### Open Field Test

The aim is to observe the locomotion and exploratory behavior of animals in the open field apparatus, similar to that described by an early study [64]. The field is manufactured of polyethylene measuring 30 cm × 30 cm, and the floor (50 cm × 50 cm) has 12 equal squares. The groups Vehicle (control group: Tween 80 0.5%), diazepam (DZP) (1 mg/kg i.p.), and ME (25, 50 and 100 mg/kg i.p.) had their substances administered 30 (DZP) and 45 (ME) minutes before the beginning of the test. The mice were positioned in the central region of the arena, and the number of animal crossings in the quadrants, dwell time in the center of the arena, amount of lifting (rearing), and latency to 1st rearing were observed for 5 min. The dwell time in the central area (4 central quadrants) was considered a measure of anxiety. We cleaned the base of the arena with 10% ethanol between tests to avoid the effects of odors left by previous subjects.

##### Investigation of the Mechanisms of Action of the Antidepressant Activity of Methyleugenol

The dose of 50 mg/kg of methyleugenol was used because of its good performance in behavioral tests. The experimental groups received dexamethasone (64 μg/kg s.c.) about 3 h 30 min before the beginning of the evaluation; we chose the tail suspension test for this step.

To ascertain the participation of the noradrenergic system in the antidepressant activity of methyleugenol, Prazosin, an α1 receptor antagonist [53,55], was used. Afterward, the different groups received the following treatments: vehicle (Tween 80 1% i.p.), ME (50 mg/kg i.p.), or prazosin (1 mg/kg i.p.). The other group received ME administration 15 min after the prazosin application. After 45 min, we performed the tail suspension test.

To investigate the serotoninergic pathway, p-chlorophenylalanine (PCPA 100 mg/kg i.p.), an inhibitor of tryptophan hydroxylase-serotonin synthesis [55], was used. We also organized two other groups that received the administration of PCPA daily for 5 days; thus, on the day of the experiment, 30 min prior to the test, one group received the methyleugenol (50 mg/kg i.p.) and the other group the saline. After 30 min, we submitted the animals to the tail suspension test.

We used SCH23390 (a D1 receptor antagonist) to investigate the dopaminergic system [35]. The groups used received vehicle (Tween 80 1% i.p.), methyleugenol (50 mg/kg i.p.), or SCH23390 (1 mg/kg s.c.). Another group received SCH23390, and after 15 min, received methyleugenol. After 30 min, we started the tail suspension test.

### 4.4. Video Recording and Analysis

All behavioral tests were engraved, and experienced researchers analyzed the behaviors in a double-blind scheme.

### 4.5. Statistical Analysis

The results obtained during the in vivo experiments passed the Shapiro–Wilk normality test. Parametric tests were used, as the data showed normal distribution. Differences for single-factor group data were ruled out by one-way analysis of variance (ANOVA) followed by Dunnett or Tukey (post hoc). Data were expressed as mean ± SEM (standard error of the mean). A 5% confidence level was adopted. All data analyses were performed with GraphPad Prism 9.0 free version (GraphPad Software, San Diego, CA, USA).

### 4.6. In Silico Methodology

#### Investigation of the Mechanisms of Action of the Antidepressant Activity of Methyleugenol

We performed molecular docking analysis to investigate the mechanisms of the action of methyleugenol via D1 dopaminergic, α1 adrenergic, and tryptophan hydroxylase receptors with antidepressant effects. Both proteins in the complex with the catechol agonist for D1 dopaminergic and inverse agonist for α1 adrenergic and inhibitor LP533401 for tryptophan hydroxylase were obtained from the Protein Data Bank (PDB) at www.rcsb.org (accessed on 1 October 2023): D1 dopaminergic (PDB ID 7CKW, ligand fenoldopam) [65] α1 adrenergic (PDB ID 7B6W, ligand (+)-Cyclazosin) [66] and tryptophan hydroxylase (PDB ID 7ZIK) [49].

We employed redocking as an assessment technique for the docking protocol. In the redocking approach, we utilized the original arrangement of a crystallized ligand bound to the protein and juxtaposed it with the positioning of a ligand affixed to the active site of the identical protein through molecular docking. To gauge the efficacy, we employed the RMSD (root-mean-square deviation) metric, which measures the average spatial disparity between the crystallized ligand and the docked ligand. A docking outcome was considered acceptable if the RMSD measured up to 2.0 Å [67]. This evaluation procedure was applied to both proteins under scrutiny in the current investigation.

Methyleugenol underwent molecular docking through Molegro Virtual Docker, version 6.0.1 (MVD). During the docking procedure, elimination of all water molecules was carried out to facilitate the docking process. Preparation of enzyme and compound structures was executed using the default parameter settings within the same software package, adhering to the following configurations: ligand evaluation criteria encompassing internal electrostatics (ES), internal hydrogen bonding, Sp2-Sp2 twists—all of which were verified; a total of 10 runs were conducted; MolDock SE algorithm was employed; interactions capped at 1500; population size limited to 50; a maximum of 300 steps; a neighbor distance factor of 1.00; and retrieval of up to 5 poses. The fitting process employed a GRID with a radius of 10 Å and resolution of 0.30 to encompass the ligand-binding site across both PDB files.

The central point of the target site served as the origin for the grid box, aligning with the ligand, which was then juxtaposed against the remaining trio of proteins, utilizing RMSD as the evaluative yardstick. We generated models incorporating pertinent attributes from both ligands that bore relevance to ligand binding. Employing the MolDock scoring algorithm in conjunction with the MolDock search algorithm [68], we harnessed Molegro Virtual Docker (CLC bio Company, Aarhus, Denmark) to generate five distinct poses for each alkaloid within the active site of every individual protein. Among these, the most stable conformation, characterized by the lowest interaction energy, was cherry-picked and integrated into the Discovery Studio 2021 software for visual scrutiny [69]. By employing the MolDock scoring mechanism, we calculated the energy of the crystallized ligand for each protein. Leveraging the MolDock score algorithm, the energy of the ligand from its crystallized configuration was seamlessly converted to the energy scale employed by the algorithm.

## 5. Conclusions

The investigation into the antidepressant-like effects of methyleugenol has yielded important results. In this study utilizing female mice, methyleugenol demonstrated a significant improvement in depressive symptoms induced by Dex, without altering locomotor activity. Notably, the observed effects of methyleugenol were associated with modulation of the D1 dopaminergic, α1 adrenergic, and serotoninergic systems.

This suggests a potential therapeutic value of methyleugenol in mitigating depres-sion-related behaviors in model animals. While these findings hold promise, further research is needed to elucidate the precise molecular mechanisms of methyleugenol’s antidepressant action, and explore potential interactions with existing antidepressant treatments.

## Figures and Tables

**Table 1:** The MolDock score and the RMSD of the docking molecular.

Molecules	D1 Dopaminergic		α1 Adrenergic		Tryptophan Hydroxylase	
	Kcal/mol	Stand. ^1^	Kcal/mol	Stand.	Kcal/mol	Stand.
Methyleugenol	−25.1705	−0.1412	−63.7474	−0.3576	−76.295	−0.428
Prazosin ^2^	-	-	−120.102	−0.3132	-	-
(+)-Cyclazosin ^3^	-	-	−135.19	−0.309	-	-
SCH 23390 ^4^	−31.5101	−0.11	-	-	-	-
Fenoldopam ^5^	−85.5469	−0.2798	-	-	-	-
PCPA ^6^	-	-	-	-	−82.953	−0.4155
LP533401 ^7^	-	-	-	-	−130.797	−0.2484
RMSD	0.19		0.3		0.6	

^1^ standardization of energies by the molar mass of each molecule; ^2^ α1 adrenergic antagonist control; ^3^ α1 adrenergic inverse agonist (PDB ligand); ^4^ D1 dopaminergic antagonist control; ^5^ D1 dopaminergic agonist (PDB ligand); ^6^ tryptophan hydroxylase inhibit control; ^7^ tryptophan hydroxylase inhibit (ligand PDB).

## Data Availability

Data is contained within the article.

## References

[1] Pitsillou E., Bresnehan S.M., Kagarakis E.A., Wijoyo S.J., Liang J., Hung A., Karagiannis T.C. (2020). The cellular and molecular basis of major depressive disorder: Towards a unified model for understanding clinical depression. Mol. Biol. Rep..

[2] Abdel-Bakky M.S., Amin E., Faris T.M., Abdellatif A.A.H. (2021). Mental depression: Relation to different disease status, newer treatments and its association with COVID-19 pandemic (Review). Mol. Med. Rep..

[3] Vitale G., Salvioli S., Franceschi C. (2013). Oxidative stress and the ageing endocrine system. Nat. Rev. Endocrinol..

[4] Borah L., Lahkar M., Dasgupta S. (2018). Study of the antidepressant activity of folic acid and vitamin-D on reserpine induced depression in mice. Asian J. Pharm. Clin. Res..

[5] Dong H., Gao Z., Rong H., Jin M., Zhang X. (2014). β-Asarone Reverses Chronic Unpredictable Mild Stress-Induced Depression-Like Behavior and Promotes Hippocampal Neurogenesis in Rats. Molecules.

[6] Mao Q.Q., Huang Z., Zhong X.M., Xian Y.F., Ip S.P. (2014). Brain-derived neurotrophic factor signalling mediates the antidepressant-like effect of piperine in chronically stressed mice. Behav. Brain Res..

[7] Sterner E.Y., Kalynchuk L.E. (2010). Behavioral and neurobiological consequences of prolonged glucocorticoid exposure in rats: Relevance to depression. Prog. Neuro-Psychopharmacol. Biol. Psychiatry.

[8] Bao A.-M., Swaab D.F. (2010). Corticotropin-Releasing Hormone and Arginine Vasopressin in Depression: Focus on the Human Postmortem Hypothalamus. Vitamins and Hormones.

[9] Holzmann I., Da Silva L.M., Corrêa Da Silva J.A., Steimbach V.M.B., De Souza M.M. (2015). Antidepressant-like effect of quercetin in bulbectomized mice and involvement of the antioxidant defenses, and the glutamatergic and oxidonitrergic pathways. Pharmacol. Biochem. Behav..

[10] Bai Y., Song L., Dai G., Xu M., Zhu L., Zhang W., Jing W., Ju W. (2018). Antidepressant effects of magnolol in a mouse model of depression induced by chronic corticosterone injection. Steroids.

[11] Fasipe O.J. (2019). The emergence of new antidepressants for clinical use: Agomelatine paradox versus other novel agents. IBRO Rep..

[12] Khushboo S.B., Sharma B. (2017). Antidepressants: Mechanism of Action, Toxicity and Possible Amelioration. J. Appl. Biotechnol. Bioeng..

[13] Khawam E.A., Laurencic G., Malone D.A. (2006). Side effects of antidepressants: An overview. Clevel. Clin. J. Med..

[14] Otte C., Gold S.M., Penninx B.W., Pariante C.M., Etkin A., Fava M., Mohr D.C., Schatzberg A.F. (2016). Major depressive disorder. Nat. Rev. Dis. Prim..

[15] Lahlou M. (2007). Screening of natural products for drug discovery. Expert Opin. Drug Discov..

[16] Zhang L., Liu J., Ge Y., Liu M. (2019). Ginkgo biloba extract reduces hippocampus inflammatory responses, improves cardiac functions and depressive behaviors in a heart failure mouse model. Neuropsychiatr. Dis. Treat..

[17] Chan E.W.C., Wong S.K., Chan H.T. (2017). Alpinia zerumbet, a ginger plant with a multitude of medicinal properties: An update on its research findings. J. Chin. Pharm. Sci..

[18] Galdino P.M., Carvalho A.A.V., Florentino I.F., Martins J.L.R., Gazola A.C., De Paula J.R., De Paula J.A.M., Torres L.M.B., Costa E.A., De Lima T.C.M. (2015). Involvement of monoaminergic systems in the antidepressant-like properties of Lafoensia pacari A. St. Hil. J. Ethnopharmacol..

[19] Hassanzadeh S.-A., Abbasi-Maleki S., Mousavi Z. (2022). Anti-depressive-like effect of monoterpene trans-anethole via monoaminergic pathways. Saudi J. Biol. Sci..

[20] Özcan M., Chalchat J.-C. (2002). Essential oil composition of *Ocimum basilicum* L. and *Ocimum minimum* L. in Turkey. Czech J. Food Sci..

[21] Lima R.K., Cardoso M.D.G., Moraes J.C., Carvalho S.M., Melo B.A., Vieira S.S. (2014). Composição química e toxicidade de óleos essenciais para o pulgão-verde Schizaphis graminum (Rondani, 1852). Arq. Inst. Biol..

[22] Irie Y., Itokazu N., Anjiki N., Ishige A., Watanabe K., Keung W.M. (2004). Eugenol exhibits antidepressant-like activity in mice and induces expression of metallothionein-III in the hippocampus. Brain Res..

[23] Lima C.C., Criddle D.N., Coelho-De-Souza A.N., Monte F.J.Q., Jaffar M., Leal-Cardoso J.H. (2000). Relaxant and antispasmodic actions of methyleugenol on guinea-pig isolated ileum. Planta Med..

[24] Tang F., Chen F., Ling X., Huang Y., Zheng X., Tang Q., Tan X. (2015). Inhibitory effect of methyleugenol on IgE-mediated allergic inflammation in RBL-2H3 cells. Mediat. Inflamm..

[25] Norte M.C.B., Cosentino R.M., Lazarini C.A. (2005). Effects of methyl-eugenol administration on behavioral models related to depression and anxiety, in rats. Phytomedicine.

[26] Farzaei M.H., Bayrami Z., Farzaei F., Aneva I., Das S.K., Patra J.K., Das G., Abdollahi M. (2020). Poisoning by Medical Plants. Arch. Iran. Med..

[27] Lukas G., Brindle S.D., Greengard P. (1971). The route of absorption of intraperitoneally administered compounds. J. Pharmacol. Exp. Ther..

[28] Nguyen E.T., Caldwell J.L., Streicher J., Ghisays V., Balmer N.J., Estrada C.M., Solomon M.B. (2018). Differential effects of imipramine and CORT 118335 (Glucocorticoid receptor modulator/mineralocorticoid receptor antagonist) on brain-endocrine stress responses and depression-like behavior in female rats. Behav. Brain Res..

[29] Canet G., Chevallier N., Zussy C., Desrumaux C., Givalois L. (2018). Central Role of Glucocorticoid Receptors in Alzheimer’s Disease and Depression. Front. Neurosci..

[30] Gregus A., Wintink A.J., Davis A.C., Kalynchuk L.E. (2005). Effect of repeated corticosterone injections and restraint stress on anxiety and depression-like behavior in male rats. Behav. Brain Res..

[31] Wróbel A., Serefko A., Piotr Wlaź E.P. (2014). The depressogenic-like effect of acute and chronic treatment with 2 dexamethasone and its influence on the activity of antidepressant drugs 3 in the forced swim test in adult mice. Prog. Neuropsychopharmacol. Biol. Psychiatry.

[32] Conti M., Spulber S., Raciti M., Ceccatelli S. (2017). Depressive-like phenotype induced by prenatal dexamethasone in mice is reversed by desipramine. Neuropharmacology.

[33] Carneiro C.A., Santos A.M.F., Guedes É.C., Cavalcante I.L., Santos S.G., Oliveira A.M.F., Barbosa F.F., Silva M.S., Almeida R.N., Salvadori M.S. (2022). Unpredictable Subchronic Stress Induces Depressive-Like Behavior: Behavioral and Neurochemical Evidences. Psychol. Neurosci..

[34] Castagné V., Moser P., Roux S., Porsolt R.D. (2011). Rodent models of depression: Forced swim and tail suspension behavioral despair tests in rats and mice. Curr. Protoc. Neurosci..

[35] Can Ö.D., Turan N., Özkay Ü.D., Öztürk Y. (2017). Antidepressant-like effect of gallic acid in mice: Dual involvement of serotonergic and catecholaminergic systems. Life Sci..

[36] Willner P., Mitchell P.J. (2002). The validity of animal models of predisposition to depression. Behav. Pharmacol..

[37] Holmes P. (2003). Rodent Models of Depression: Reexamining Validity Without Anthropomorphic Inference. Crit. Rev. Neurobiol..

[38] Castagné V., Porsolt R.D., Moser P. (2009). Use of latency to immobility improves detection of antidepressant-like activity in the behavioral despair test in the mouse. Eur. J. Pharmacol..

[39] Sigwalt A.R., Budde H., Helmich I., Glaser V., Ghisoni K., Lanza S., Cadore E.L., Lhullier F.L.R., de Bem A.F., Hohl A. (2011). Molecular aspects involved in swimming exercise training reducing anhedonia in a rat model of depression. Neuroscience.

[40] Wróbel A., Serefko A., Wlaź P., Poleszak E. (2015). The effect of imipramine, ketamine, and zinc in the mouse model of depression. Metab. Brain Dis..

[41] Moretti M., Colla A., De Oliveira Balen G., Dos Santos D.B., Budni J., De Freitas A.E., Farina M., Severo Rodrigues A.L. (2012). Ascorbic acid treatment, similarly to fluoxetine, reverses depressive-like behavior and brain oxidative damage induced by chronic unpredictable stress. J. Psychiatr. Res..

[42] Zeni A.L.B., Camargo A., Dalmagro A.P. (2017). Ferulic acid reverses depression-like behavior and oxidative stress induced by chronic corticosterone treatment in mice. Steroids.

[43] de Souza I.B.M.B., Costa L.R.F., Tiago P.R.F., Cagni F.C., Lima R.H., Silva Junior E.D., Gavioli E.C. (2019). Venlafaxine and Nortriptyline Reverse Acute Dexamethasone-Induced Depressive-Like Behaviors in Male and Female Mice. Exp. Clin. Psychopharmacol..

[44] Rodrigues A.L.S., Rocha J.B.T., Mello C.F., Souza D.O. (1996). Effect of perinatal lead exposure on rat behaviour in open-field and two-way avoidance tasks. Pharmacol. Toxicol..

[45] Choleris E., Thomas A.W., Kavaliers M., Prato F.S. (2001). A detailed ethological analysis of the mouse open field test: Effects of diazepam, chlordiazepoxide and an extremely low frequency pulsed magnetic field. Neurosci. Biobehav. Rev..

[46] Prut L., Belzung C. (2003). The open field as a paradigm to measure the effects of drugs on anxiety-like behaviors: A review. Eur. J. Pharmacol..

[47] Ge H., Bian Y., He X., Xie X.Q., Wang J. (2019). Significantly different effects of tetrahydroberberrubine enantiomers on dopamine D1/D2 receptors revealed by experimental study and integrated in silico simulation. J. Comput. Aided. Mol. Des..

[48] Qian W., Lu W., Sun H., Li Z., Zhu L., Zhao R., Zhang L., Zhou S., Zhou Y., Jiang H. (2012). Design, synthesis, and pharmacological evaluation of novel tetrahydroprotoberberine derivatives: Selective inhibitors of dopamine D1 receptor. Bioorg. Med. Chem..

[49] Specker E., Matthes S., Wesolowski R., Schütz A., Grohmann M., Alenina N., Pleimes D., Mallow K., Neuenschwander M., Gogolin A. (2022). Structure-Based Design of Xanthine-Benzimidazole Derivatives as Novel and Potent Tryptophan Hydroxylase Inhibitors. J. Med. Chem..

[50] Brunello N., Blier P., Judd L.L., Mendlewicz J., Nelson C.J., Souery D., Zohar J., Racagni G. (2003). Noradrenaline in mood and anxiety disorders: Basic and clinical studies. Int. Clin. Psychopharmacol..

[51] Kitada Y., Miyauchi T., Kanazawa Y., Nakamichi H., Satoh S. (1983). Involvement of α- and β1-adrenergic mechanisms in the immobility-reducing action of desipramine in the forced swimming test. Neuropharmacology.

[52] Masuda Y., Ohnuma S., Sugiyama T. (2001). α2-Adrenoceptor activity induces the antidepressant-like glycolipid in mouse forced swimming. Methods Find. Exp. Clin. Pharmacol..

[53] Chellian R., Pandy V., Mohamed Z. (2016). Biphasic effects of α-asarone on immobility in the tail suspension test: Evidence for the involvement of the noradrenergic and serotonergic systems in its antidepressant-like activity. Front. Pharmacol..

[54] Cryan J.F., Valentino R.J., Lucki I. (2005). Assessing substrates underlying the behavioral effects of antidepressants using the modified rat forced swimming test. Neurosci. Biobehav. Rev..

[55] Abbasi-Maleki S., Mousavi Z. (2017). Hydroethanolic extract of Carthamus tinctorius induces antidepressant-like effects: Modulation by dopaminergic and serotonergic systems in tail suspension test in mice. Iran. J. Basic Med. Sci..

[56] Berridge K.C. (2018). Evolving concepts of emotion and motivation. Front. Psychol..

[57] Millan M.J., Lejeune F., Gobert A., Brocco M., Auclair A., Bosc C., Rivet J.M., Lacoste J.M., Cordi A., Dekeyne A. (2000). S18616, a highly potent spiroimidazoline agonist at alpha(2)-adrenoceptors: II. Influence on monoaminergic transmission, motor function, and anxiety in comparison with dexmedetomidine and clonidine. J. Pharmacol. Exp. Ther..

[58] Naranjo C.A., Tremblay L.K., Busto U.E. (2001). The role of the brain reward system in depression. Prog. Neuro-Psychopharmacol. Biol. Psychiatry.

[59] Lehr E. (2002). Potential antidepressant properties of pramipexole detected in locomotor and operant behavioral investigations in mice. Psychopharmacology.

[60] Protti M., Mandrioli R., Marasca C., Cavalli A., Serretti A., Mercolini L. (2020). New-generation, non-SSRI antidepressants: Drug-drug interactions and therapeutic drug monitoring. Part 2: NaSSAs, NRIs, SNDRIs, MASSAs, NDRIs, and others. Med. Res. Rev..

[61] ANVISA ANVISA—Agência Nacional de Vigilância Sanitária. https://portal.anvisa.gov.br/wps/portal/anvisa/home.

[62] OECD (2001). The Organization of Economic Co-Operation and Development Guidelines Test No. 423: Acute Oral Toxicity—Acute Toxic Class Method, OECD Guidelines for the Testing of Chemicals, Section 4.

[63] Thierry B., Simon P., Porsolt R.D. (1986). Psychopharmacology The tail suspension test: Ethical considerations. Psychopharmacology.

[64] Broadhurst P.L. (1960). Experiments in psychogenetics. Experiments in Personality.

[65] Teng X., Chen S., Nie Y., Xiao P., Yu X., Shao Z., Zheng S. (2022). Ligand recognition and biased agonism of the D1 dopamine receptor. Nat. Commun..

[66] Deluigi M., Morstein L., Schuster M., Klenk C., Merklinger L., Cridge R.R., de Zhang L.A., Klipp A., Vacca S., Vaid T.M. (2022). Crystal structure of the α1B-adrenergic receptor reveals molecular determinants of selective ligand recognition. Nat. Commun..

[67] Onodera K., Satou K., Hirota H. (2007). Evaluations of molecular docking programs for virtual screening. J. Chem. Inf. Model..

[68] Thomsen R., Christensen M.H. (2006). MolDock: A new technique for high-accuracy molecular docking. J. Med. Chem..

[69] Dassault Systèmes (2023). BIOVIA Discovery Studio Client, Version BIOVIA 2021. San Diego: Dassault Systèmes. https://discover.3ds.com/discovery-studio-visualizer-download.

